# Universal Actuation Module and Kinematic Model for Heart Valve Interventional Catheter Robotization

**DOI:** 10.1109/LRA.2024.3388789

**Published:** 2024-06

**Authors:** Weizhao Wang, Zicong Wu, Carlo Saija, Aya Zeidan, Zhouyang Xu, Aryana Pishkahi, Tiffany Patterson, Simon Redwood, Shuangyi Wang, Kawal Rhode, Richard Housden

**Affiliations:** 1 School of Biomedical Engineering and Imaging Sciences, King’s College London, WC2R 2LS London, U.K.; 2 Cardiovascular Department, Guy’s and St Thomas’ NHS Foundation Trust, SE1 7EH London, U.K.; 3 School of Cardiovascular Medicine and Sciences, King’s College London, SE1 7EH London, U.K.; 4 State Key Laboratory of Multimodal Artificial Intelligence Systems, Institute of Automation, Chinese Academy of Sciences, Beijing 100190, China; School of Artificial Intelligence, University of Chinese Academy of Sciences, Beijing 101408, China; Centre for Artificial Intelligence and Robotics, Hong Kong Institute of Science & Innovation, Chinese Academy of Sciences, Hong Kong

**Keywords:** Kinematics, actuation and joint mechanisms, modeling, control, learning for soft robots

## Abstract

Catheters have been widely used to deal with heart valve diseases. However, the diversity in handle structures and bending curvatures imposes significant complexities in safe delivery and positioning. In this work, we designed a module for single knob actuation assembled coaxially on the catheter handle, composed of a chuck for universal clamping of diameters from 15 to 45 mm and a position-adjustable shaft to accommodate various spacing between knobs. In addition, we proposed a two-curvature with pseudo joints (TC-PJ) model for bending control of bendable sections (BSs) in catheters. The verification was decoupled into two steps based on the other three deformation patterns. Firstly, comparing the two-curvature (TC) model with pseudo-rigid-body (PRB), constant curvature (CC), and Euler spiral (ES) models to simulate planar bending and elongation, the results showed a more accurate shape representation. Then, five distinct catheters were employed to test the clamping universality of the module and tip positioning precision of the TC-PJ model which took torsion and shear strain into consideration. The root-mean-square error (RMSE) and the standard deviation (SD) of tip position and direction were analysed. Results indicated the module’s suitability for clamping these catheters, with the large guide sheath exhibiting minimal position RMSE (SD) of around 0.10 (0.051) mm and 0.049 (2.15) degrees, while the puncture catheter demonstrated the highest position and direction RMSE (SD) extending to about 1.16 (0.53) mm and 0.70 (31.33) degrees, primarily attributed to the coupling of two sequential bendable components. Overall, the proposed actuation module and kinematic model showed the ability of universal manipulation and an average tip position and direction RMSE of 0.65 mm and 0.23 degrees in free space.

## 1 Introduction

**Fig. 1:**
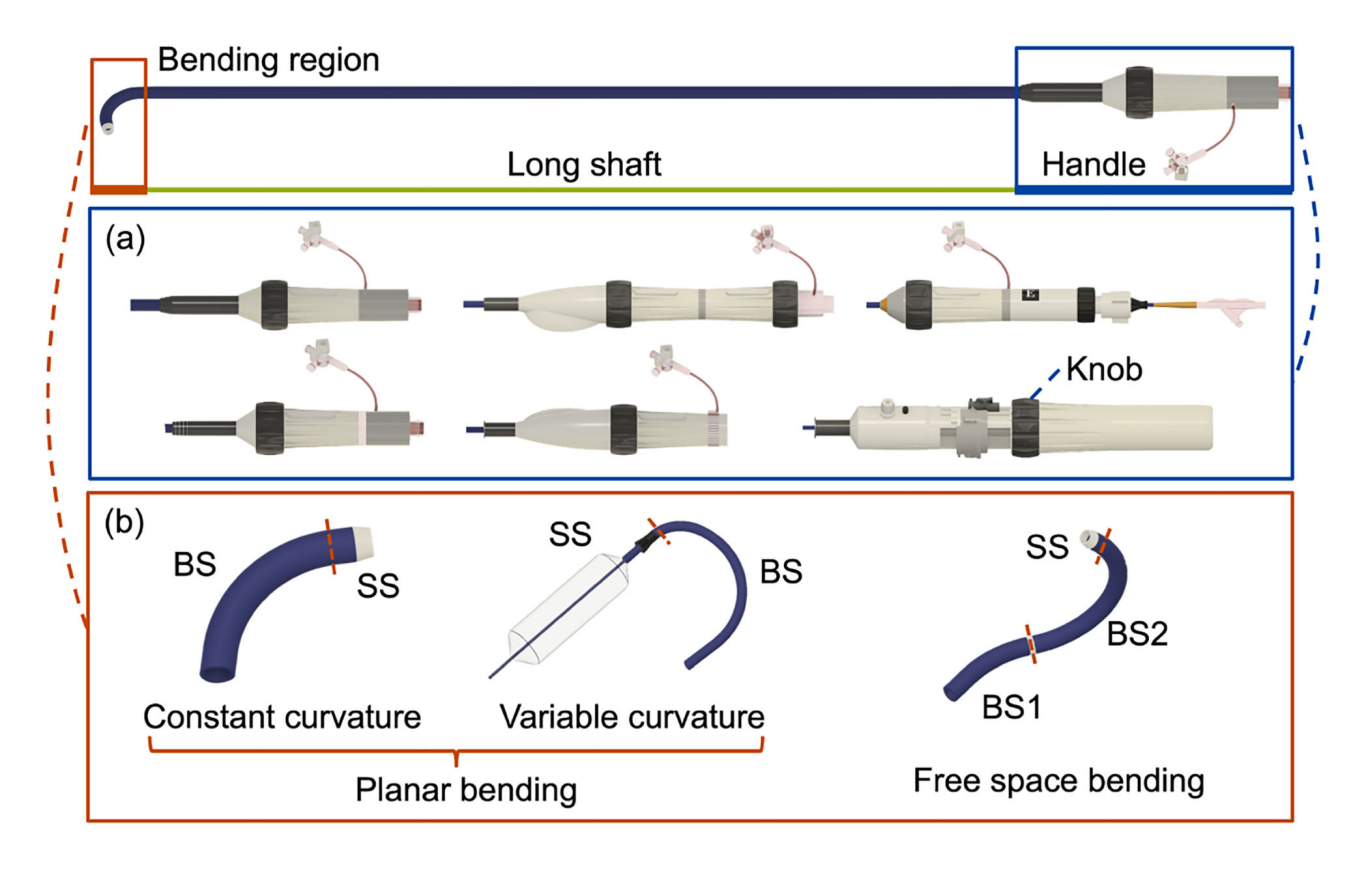
Structure of commonly used catheters for valve interventions.

TRANSCATHETER heart valve interventions have emerged over the past decade and have introduced a pivotal transformation in the therapy of heart valve diseases [1]. Moreover, technological improvements have facilitated the expansion of these interventions to younger or lower-risk patients [2]. However, the interventional catheter composed of a handle, a long shaft, and a bending region (top graph in Fig. 1), faces challenges in navigating narrow anatomical pathways [3] and aligning with the native valve in the dynamic environment of the heart [4], [5] due to its inherent flexibility and nonlinearity [6]. Successful manipulation of the catheter also depends on the skill level and fatigue of the cardiologist [7], [8]. Meanwhile, the procedures are typically conducted under the guidance of real-time fluoroscopy [9]. To mitigate these challenges, a robotic system for catheter manipulation would not only improve catheter positioning but also protect cardiologists from potentially harmful radiation exposure [10], [11].

Furthermore, commercial catheters for valve repair or replacement are diverse [2], [12] and the handle structures are equipped with at least one knob. The knob varies in diameter and spacing, and is commonly positioned either coaxially (more prevalent) or laterally with the handle (Fig. 1a). A bending region is attached at a long shaft’s end, composed of single or multiple planar bendable sections (BSs) for creating a bending pathway for the delivery and one straight section (SS) for medical device attachment (Fig. 1b). Each BS is typically laser cut to include a number of circumferential slots [13]. Constant curvature is attained through slots of uniform width, while variable curvature relies on slots of varying widths. Additionally, the bending region can navigate through free space by combining multiple BSs orthometrically. Each BS is individually controlled by a dedicated knob. The manipulation of diverse handles and spatial understanding of various bending shapes are quite challenging for the operator and important for the safety of delivery. Consequently, the universality of the robotic system for catheter actuation, bending control, and shape modeling would confer a significant advantage to cardiologists.

There are few universal actuation systems for interventional catheter manipulation. Zhang [10] developed a robotic system to help with valve edge-to-edge repair, but it was specifically designed to actuate and control the MitraClip^*TM*^. Norouzi optimized an automated catheter operating system with an inclusive structural design, to manipulate different catheters by replacing compartments. Nevertheless, this approach introduced an additional operational step of interchanging compartments when transitioning between different catheters. To achieve universal actuation, we designed an actuation module for single knob (coaxially assembled) manipulation incorporating a three-jaw chuck for universal clamping and a sliding shaft to deal with various spacing between knobs. A catheter handle can be actuated by combining multiple modules. This work is detailed in Section II-A.

**Fig. 2:**
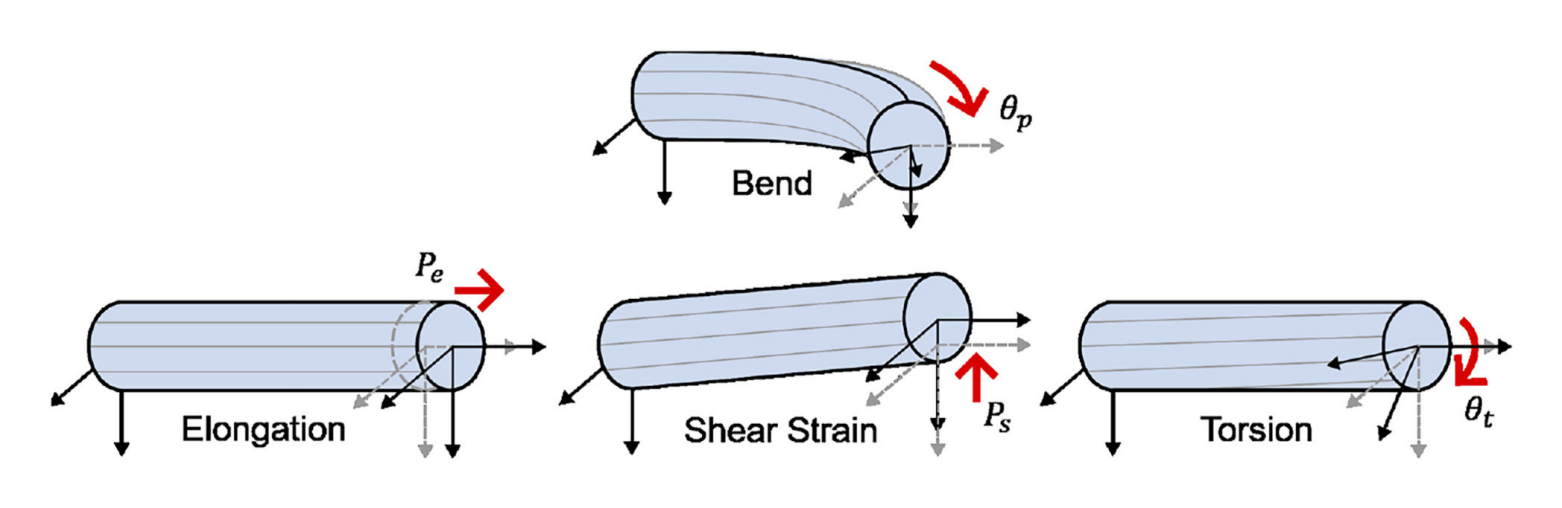
Four deformations of a BS: bending, elongation, shear strain, and torsion.

To establish a universal mapping from the rotation of the actuation module to the shape and position of relevant BS for bending control, we aimed to bypass reliance on catheter structural and material properties. We also tried to consider the other three deformation patterns (elongation, torsion, shear strain) [15] of a BS (Fig. 2) for precise positioning while planar bending. There are four common methodologies for continuum body modeling by the latest review [16]. Cosserat rod theory, which is a mathematical framework used to model slender structures characterized by intricate deformation behaviours, was employed to model MitraClip^*TM*^ in [10]. However, it is based on known structural characteristics and material properties intrinsic to catheters. Surrogate models, also known as data-driven methods, require a large amount of data for training and are inconvenient to apply to different catheters. The acquisition of sufficiently representative data across diverse catheters can be resource-intensive and may not always be readily attainable. In this work, we proposed a two-curvature with pseudo joints (TC-PJ) model and employed regression methods to calibrate the model parameters for mapping. The modeling process can be decoupled into two steps. Initially, a two-curvature (TC) model was used to simulate variable curvature for planar bending and elongation by combining two groups of slots with different widths in series. The curvature of the BS can also be constant once the widths are the same. There are four degrees of freedom (DoFs) in this model which can achieve planar positioning with three DoFs and shape adjustment with one extra DoF. More than two curvatures in this model will increase the complexity of modeling and latency (time-consuming calculation) of control. Subsequently, pseudo joints (PJ) were added to simulate shearing and twisting independently. Specifically, two rotation joints at both ends of the BS are to simulate the torsion along the BS. A translational joint along the BS is to simulate the shear strain. Further details on this approach are provided in Section II-B. In Section II-C, the testing was also divided into two steps. Firstly, the TC model was applied to simulate random planar bending and elongation of the BS and compare it with the commonly used planar bending models, such as pseudo-rigid-body (PRB), constant curvature (CC), and Euler spiral (ES) models in positioning and shape representation. Then, we established an experimental platform to verify the universality of the actuation module and tip positioning precision of the TC-PJ model. The results are presented in Section III. Section IV discusses, concludes the work, and proposes some future work on catheter robot design and control.

Our contributions are as follows: 1) this customized actuation module can securely grip and control a diverse range of catheter knobs with diameters below 45 mm; 2) the proposed TC model is more accurate than the PRB, CC, and ES models to simulate the random bending patterns of a BS within a plane; 3) the TC-PJ model shows universal applicability across various catheters for tip positioning in free space.

## II Methods

### A Universal Actuation Module Design

The design rationale of robotic systems for catheter actuation typically imitates the manual manipulation of catheters by the cardiologist, which is composed of clamping, translational, and rotational mechanisms. In the context of these robotic systems, the latter two mechanisms exhibit universality across different implementations. However, clamping mechanisms are different because of the various catheter structures. Furthermore, the difference is mainly embodied in various knobs and handles. The knobs of the majority of catheters are coaxially assembled with the handles. The objective of this subsection is to design an actuation module with a small footprint to accommodate a coaxially assembled knob or handle.

#### 1) Clamping Mechanism Design

**Fig. 3:**
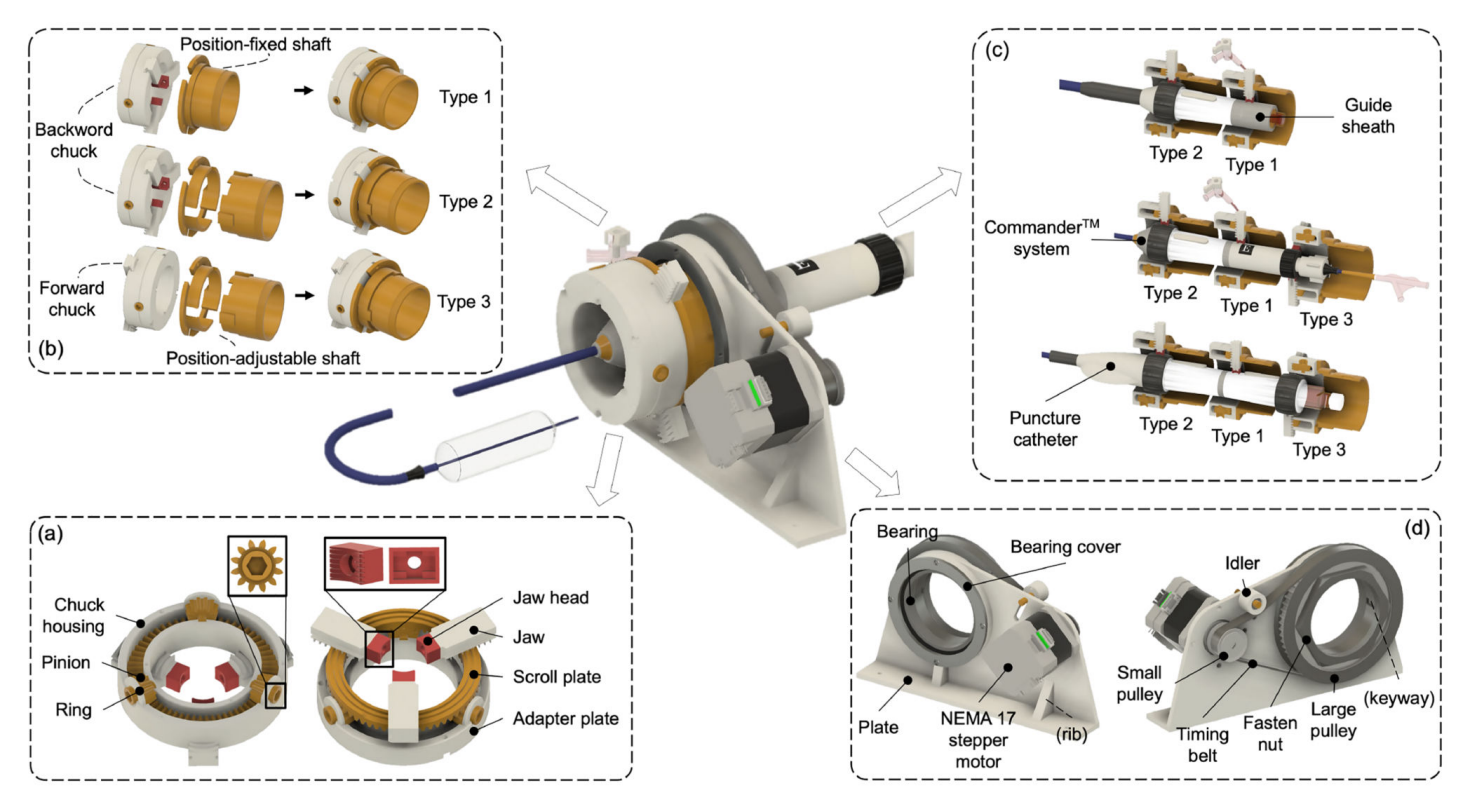
Design of a universal actuation module. (a) The pinion features a hexagonal hole for electric gripping, and a high-elasticity rubber material serves as the jaw head to enhance grip stability and prevent damage. (b) Two types of shaft, one with a fixed position and the other adjustable, are designed for transmitting rotational motion. The chuck assembly offers two orientations: forward and backward. (c) Clamping performance testing is conducted using a guide sheath, Commander^*TM*^ system, and puncture catheter. (d) The module is enclosed within a bearing and plate housing and operates through a timing belt transmission.

The lathe chuck, a fundamental tool in machining for secure workpiece rotation, is also suitable for clamping knobs assembled coaxially with the handle. We customized a three-jaw chuck with a 50 mm inner diameter based on the range of knob sizes (from 15 to 45 mm) and an 80 mm outer diameter for compactness (Fig. 3a). Self-centering is facilitated by a scroll plate (pitch 3 mm, teeth 62), and clamping or releasing is achieved through the rotation of three pinions (teeth 12). For clamping efficiency, the pinion was designed with a hexagon hole (for size 5 mm hex wrench) which can accommodate electric screwdrivers. In addition, we used F80 resin material (RESIONE) which is soft and elastic for the manufacturing of jaw heads to prevent slipping and clamping damage, and to make sure that the jaws were gripping the knob evenly to avoid any wobbling or vibration during the intervention. Each jaw has two contact points with the knob, enhancing stability and concentricity.

Two types of shafts, position-fixed and position-adjustable, were developed to connect with the chuck for transmitting rotational motion (Fig. 3b). The position-adjustable chuck, with grooves of 6 mm length for axial adjustment, accommodates knobs with variable spacing. The shaft can be assembled on either side of the chuck to prevent interference with other chucks and the catheter. To clamp an entire catheter, a position-fixed chuck (Type 1) secures the handle, while position-adjustable chucks (Type 2 or 3) are used for knob clamping, ensuring adaptability across different catheters (Fig. 3c).

#### 2) Housing and Actuation Design

The housing structure pictured in Fig. 3d was constructed to actuate the chuck with one DoF. To accomplish this controlled movement, a NEMA 17 stepper motor (0.4 Nm Torque, 1.8°, 42.3 x 42.3 mm Frame, 5mm Shaft) was employed as the driving force. The rotational motion was conveyed through the implementation of a timing belt transmission system (1:5 transmission ratio). Notably, an idler component was incorporated into the system design, serving to shorten the overall width of the plate. Most structural compartments of this chuck module, including support and housing, were 3D printed using polylactic acid (PLA) materials. However, components requiring heightened strength and robust support, including pinions, jaws, the scroll plate, and pulleys, were crafted from nylon, a material known for its excellent mechanical properties.

### B Universal BS Kinematic Model

The actuation of the knob results in the bending of the connected BS as the chuck rotates. To streamline the modeling process, we initially addressed elongation in a 2D scenario and subsequently incorporated torsion and shear strain patterns in free space, effectively decoupling the analysis.

#### 1) Kinematic Model (elongation)

**Fig. 4:**
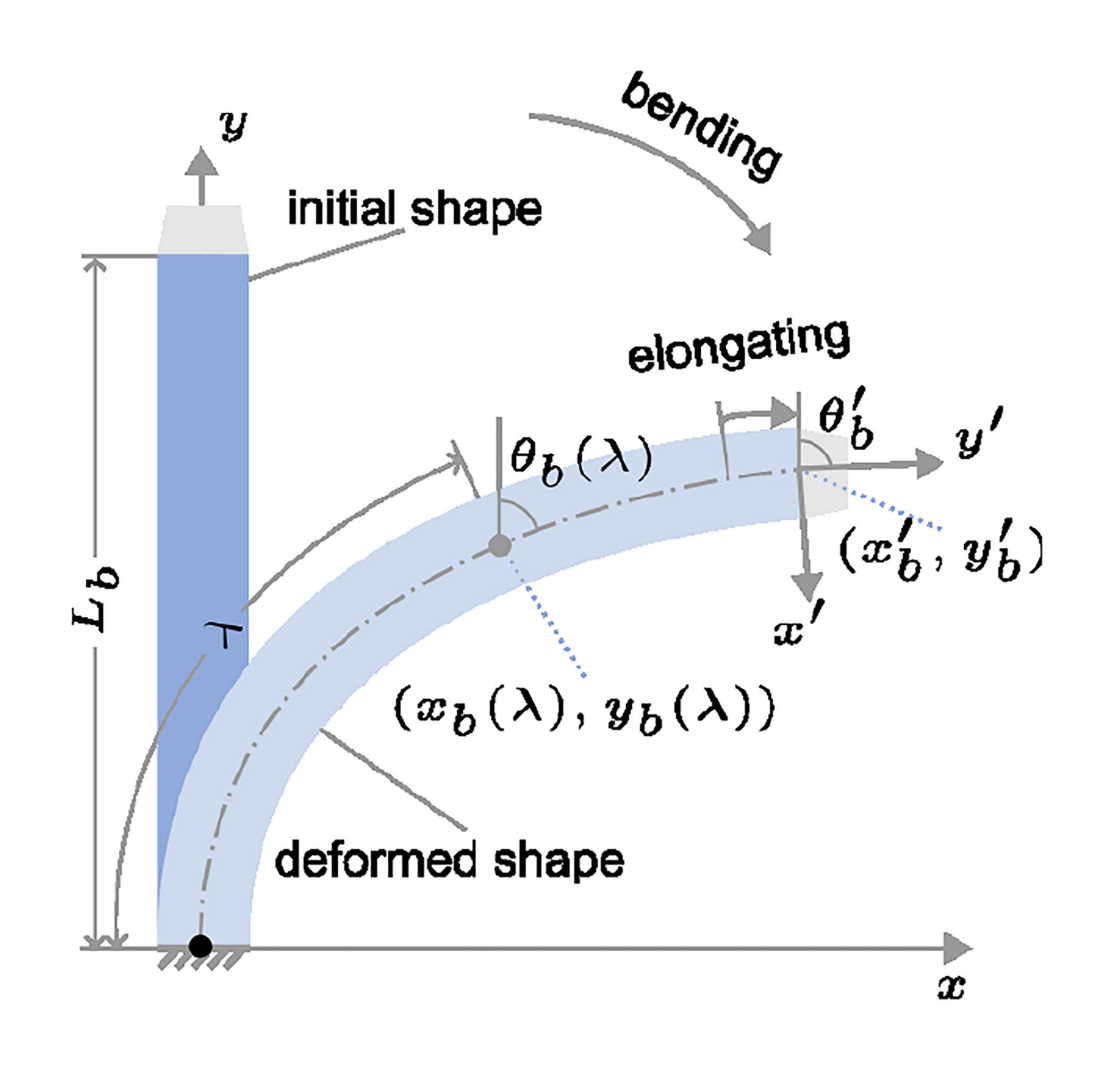
A deformed BS within a plane (including bending and elongation). *θ*_*b*_(*λ*) is the bending angle at (*x*_*b*_(*λ*), *y*_*b*_(*λ*)) with curvature length *λ*.

The model is initially defined in 2D (Fig. 4), where a BS can bend and elongate to a position $\left(x_{b}^{\prime },y_{b}^{\prime }\right)$ towards a direction (bending angle) $\theta_{b}^{\prime }$ within a plane. *L*_*b*_ represented the length of the non-deformed BS, which is assumed to be unknown in this case. A 3*3 matrix $T_{b}^{\prime }$ represents the transformation (1) from the distal end to the proximal end of the BS.

(1) $$T_{b}^{\prime }=\left[\begin{array}{ccc} c\theta_{b}^{\prime } & s\theta_{b}^{\prime } & x_{b}^{\prime }\\ -s\theta_{b}^{\prime } & c\theta_{b}^{\prime } & y_{b}^{\prime }\\ 0 & 0 & 1 \end{array}\right]$$

where s* and c* are the sine and cosine of angle (*).

**Fig. 5:**
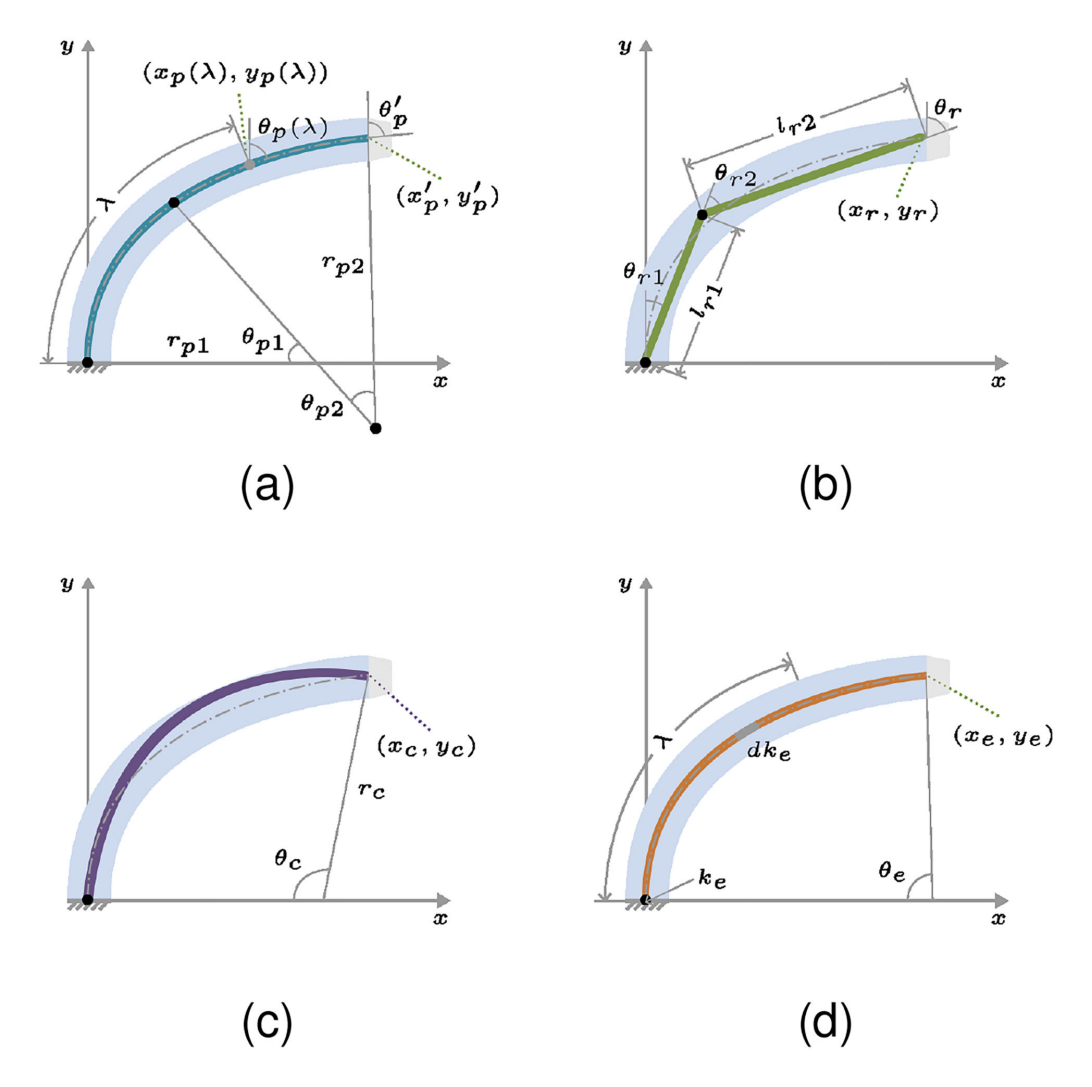
Comparison of the proposed model with three commonly used models. (a) The TC model integrates two constant curvatures in a serial arrangement. (b) The PRB model consists of two rigid links connected in series. (c) The CC model maintains a constant curvature. (d) The ES model exhibits linear changes in curvature.

Our method proposes to combine two curvatures (TC) to describe a randomly bending and elongating BS. The shape was parameterized by angle *θ*_*p*1_, radius *r*_*p*1_ of curvature1 and angle *θ*_*p*2_, radius *r*_*p*2_ of curvature2, as shown in Fig. 5a. In addition, bending angle $\theta_{p}^{\prime }=\theta_{p1}+\theta_{p2}$, length of curvature1 *l*_*p*1_ = *r*_*p*1_ * *θ*_*p*1_, and length of curvature2 *l*_*p*2_ = *r*_*p*2_ * *θ*_*p*2_ were calculated. Constant curvature theory and classic robot kinematics were used to calculate the transformation matrix $T_{p}^{\prime }$ and shape information (*x*_*p*_(*λ*), *y*_*p*_(*λ*)), as shown in (2) and (3). In addition, we used two variables *H*_1_ and *H*_2_ to simplify (3): *H*_1_ = *r*_*p*2_(*cθ*_*p*_(*λ*) − *cθ*_*p*1_), *H*_2_ = *r*_*p*2_(*sθ*_*p*_(*λ*) − *sθ*_*p*1_).

(2) $$T_{p}^{\prime }=\left[\begin{array}{ccc} c\theta_{p}^{\prime } & s\theta_{p}^{\prime } & r_{p1}\left(1-c\theta_{p1}\right)-r_{p2}\left(c\theta_{p}^{\prime }-c\theta_{p1}\right)\\ -s\theta_{p}^{\prime } & c\theta_{p}^{\prime } & r_{p1}*s\theta_{p1}+r_{p2}\left(s\theta_{p}^{\prime }-s\theta_{p1}\right)\\ 0 & 0 & 1 \end{array}\right]$$

(3) $$\begin{array}{l} x_{p}(\lambda )=\{\begin{array}{ll} r_{p1}\left(1-c\theta_{p}(\lambda )\right), & \;\text{if}\;0\leq \lambda <l_{p1}\\ r_{p1}\left(1-c\theta_{p1}\right)-H_{1}, & \;\text{if}\;l_{p1}\leq \lambda <l_{p1}+l_{p2} \end{array}\\ y_{p}(\lambda )=\{\begin{array}{ll} r_{p1}*s\theta_{p}(\lambda ), & \;\text{if}\;0\leq \lambda <l_{p1}\\ r_{p1}*s\theta_{p1}+H_{2}, & \text{if}\;l_{p1}\leq \lambda <l_{p1}+l_{p2} \end{array} \end{array}$$

Kinematic solutions were derived as (4) by matching equations (1) and (2). As for representing the shape, the Fréchet distance [17] is a measurement to quantify the similarities between two curves while taking into account their shapes. We employed the Fréchet distance *FD*(*θ*_*p*1_) to measure the difference between the random shape and the model shape. Each catheter shape was discretized into a union containing n points, $\overset{n}{\underset{i=1}{\cup }}\;\left(x_{b}(i),y_{b}(i)\right)$ and $\overset{n}{\underset{i=1}{\cup }}\;\left(x_{p}(i),y_{p}(i)\right)$. The task was to find the minimum value of (5) based on a reference angle *θ*_*ref*_. Thus the model was simplified to solve two equations:

(4) $$\{\begin{array}{l} r_{p1}\left(1-c\theta_{p1}\right)-r_{p2}\left(c\theta_{p}^{\prime }-c\theta_{p1}\right)=x_{b}^{\prime }\\ \;r_{p1}*s\theta_{p1}+r_{p2}\left(s\theta_{p}^{\prime }-s\theta_{p1}\right)=y_{b}^{\prime }\\ \;\theta_{p}^{\prime }=\theta_{b}^{\prime } \end{array}$$

(5) $$\theta_{p1}^{*}=\arg\min||FD\left(\theta_{p1}\right),\theta_{ref\;}||,\theta_{p1}\in [-pi,pi]$$

#### 2) Kinematic Model (torsion and shear strain)

**Fig. 6:**
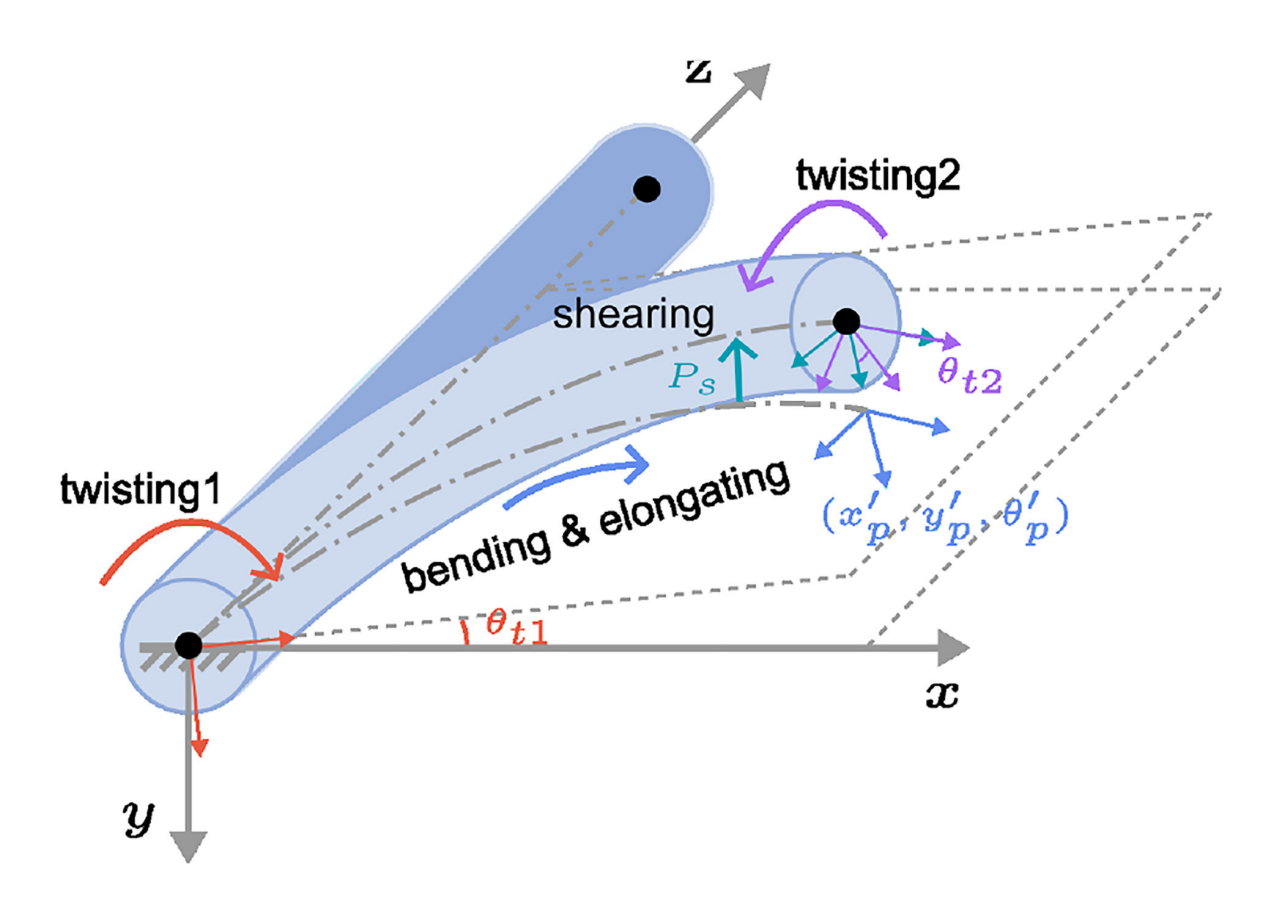
A deformed BS within free space. The variables *θ*_*t*1_, $\left(x_{p}^{\prime },y_{p}^{\prime },\theta_{p}^{\prime }\right)$, *P*_*s*_, and *θ*_*t*2_ correspond to the parameters for twisting1, deformation within a plane, shearing, and twisting2, respectively.

Upon the tip bending in free space, the additional aspects of twisting and shearing were considered within the modeling framework. To address these deformations effectively, a TC model combined with pseudo joints was introduced to emulate these mechanical responses. Specifically, rotational joints *θ*_*t*1_ and *θ*_*t*2_ were strategically positioned at both the proximal and distal ends to replicate twisting movements, while a prismatic joint *P*_*s*_ was situated at the proximal end to simulate shearing motions, as illustrated in Fig. 6. $x_{p}^{\prime },y_{p}^{\prime }\;$, and $\theta_{p}^{\prime }$ are variables in the 2D TC model. The transformation matrices of each joint are listed as (6) to (9):

(6) $$T_{t1}=\left[\begin{array}{cccc} c\theta_{t1} & s\theta_{t1} & 0 & 0\\ -s\theta_{t1} & c\theta_{t1} & 0 & 0\\ 0 & 0 & 1 & 0\\ 0 & 0 & 0 & 1 \end{array}\right]$$

(7) $$T_{TC}=\left[\begin{array}{cccc} c\theta_{p}^{\prime } & 0 & s\theta_{p}^{\prime } & x_{p}^{\prime }\\ 0 & 1 & 0 & 0\\ -s\theta_{p}^{\prime } & 0 & c\theta_{p}^{\prime } & y_{p}^{\prime }\\ 0 & 0 & 0 & 1 \end{array}\right]$$

(8) $$T_{ps}=\left[\begin{array}{cccc} 1 & 0 & 0 & 0\\ 0 & 1 & 0 & P_{s}\\ 0 & 0 & 1 & 0\\ 0 & 0 & 0 & 1 \end{array}\right]$$

(9) $$T_{t2}=\left[\begin{array}{cccc} c\theta_{t2} & s\theta_{t2} & 0 & 0\\ -s\theta_{t2} & c\theta_{t2} & 0 & 0\\ 0 & 0 & 1 & 0\\ 0 & 0 & 0 & 1 \end{array}\right]$$

(10) $$T_{TC-PJ}=\left[\begin{array}{cccc} c\theta_{t1}c\theta_{p}^{\prime }c\theta_{t2}-s\theta_{t1}s\theta_{t2} & c\theta_{t1}c\theta_{p}^{\prime }s\theta_{t2}+s\theta_{t1}c\theta_{t2} & c\theta_{t1}s\theta_{p}^{\prime } & x_{p}^{\prime }c\theta_{t1}+P_{s}s\theta_{t1}\\ -s\theta_{t1}c\theta_{p}^{\prime }c\theta_{t2}-c\theta_{t1}s\theta_{t2} & -s\theta_{t1}c\theta_{p}^{\prime }s\theta_{t2}+c\theta_{t1}c\theta_{t2} & -s\theta_{t1}s\theta_{p}^{\prime } & -x_{p}^{\prime }s\theta_{t1}+P_{s}c\theta_{t1}\\ -s\theta_{p}^{\prime }c\theta_{t2} & -s\theta_{p}^{\prime }s\theta_{t2} & c\theta_{p}^{\prime } & y_{p}^{\prime }\\ 0 & 0 & 0 & 1 \end{array}\right]$$

The construction of the mapping (10) from model parameters to BS tip transformation matrix *T*_*TC*−*PJ*_ was calculated through the amalgamation of equations (6) through (9). To achieve comprehensive kinematics from the actuation module angle *θ*_*k*_ to *T*_*TC*−*PJ*_, two intermediary mappings are crucial: one that relates *θ*_*k*_ to the tip bending angle $\theta_{p}^{\prime }$, and the other that links $\theta_{p}^{\prime }$ to the model parameters. While the specification of *θ*_*k*_ and *T*_*TC*−*PJ*_ is straightforward by collecting experimental data, the acquisition of $\theta_{p}^{\prime }$ and model parameters entails a series of steps. Firstly, the parameter *θ*_*t*1_ was determined as the inverse tangent of *T*_*TC*−*PJ*_ (1, 3) and *T*_*TC*−*PJ*_ (2, 3). Secondly, a similar methodology was applied to calculate *θ*_*t*2_. Thirdly, the transformation matrix *T*_*T* *C*_ * *T*_*ps*_ could be calculated as $T_{t1}^{-1}\left(\theta_{t1}\right)*T_{TC-PJ}*T_{t2}^{-1}\left(\theta_{t2}\right)$ and the value of *P*_*s*_ was (*T*_*T* *C*_ * *T*_*ps*_)(2, 4). Additionally, $\theta_{p}^{\prime }$ was solved based on *T*_*TC*−*PJ*_ (3, 1). Finally, the variables *θ*_*p*1_, *r*_*p*1_, *θ*_*p*2_, and *r*_*p*2_ were ascertained employing the TC model. Then polynomial regression techniques were employed to establish the nonlinear relationships among these parameters, as denoted by equations (11) and (12). In addition, the degrees of the polynomials depend on the complexity of the relationships. By integrating these equations into the framework presented in (10), we could derive a kinematic mapping from *θ*_*k*_ to *T*_*TC*−*PJ*_.

(11) $$\theta_{p}^{\prime }=f_{reg0}\left(\theta_{k}\right)$$

(12) $$\{\begin{array}{c} \theta_{t1}=f_{reg1}\left(\theta_{p}^{\prime }\right)\\ \theta_{p1}=f_{reg2}\left(\theta_{p}^{\prime }\right)\\ \theta_{p2}=\theta_{p}^{\prime }-\theta_{p1}\\ r_{p1}=f_{reg3}\left(\theta_{p}^{\prime }\right)\\ r_{p2}=f_{reg4}\left(\theta_{p}^{\prime }\right)\\ P_{s}=f_{reg5}\left(\theta_{p}^{\prime }\right)\\ \theta_{t2}=f_{reg6}\left(\theta_{p}^{\prime }\right) \end{array}$$

### C Simulation and Experimental Set-up

A random planar bending simulation environment was constructed to validate the universality and accuracy of the TC model. Subsequently, an experimental platform was developed to verify the positioning precision of the TC-PJ model.

#### 1) Random Planar Bending Simulation

The versatility of laser-cut hypotube technology enables the production of catheters with a range of curvatures by combining slots with various widths in series. Therefore, we combined four constant curvatures, where each was characterized by random angle and radius values, to simulate a complex bending and elongation BS required for realistic catheter applications. Position, direction errors, and Fréchet distance of the model were used to test the outcome.

In comparison, three commonly used continuum robot models are shown in Fig. 5b-5d. The PRB model has revolute *θ*_*r*1_, prismatic *l*_*r*1_, revolute *θ*_*r*2_, and prismatic *l*_*r*2_ joints, and bending angle is calculated as *θ*_*r*_ = *θ*_*r*1_ + *θ*_*r*2_. Its transformation matrix *T*_*r*_ is calculated as (13a). The transformation matrix of the CC model *T*_*c*_ is driven by the angle *θ*_*c*_ and radius *r*_*c*_ of curvature. As for the ES model, the transformation matrix *T*_*e*_ is parameterized by curvature at the proximal end *k*_*e*_, the rate of curvature change *dk*_*e*_, and length *l*_*e*_. In addition, bending angle is *θ*_*e*_ = − (*k*_*e*_ + *dk*_*e*_ * *l*_*e*_*/*2) * *l*_*e*_ and curvature at the distal end is *k*(*λ*) = *k*_*e*_ + *dk*_*e*_ * *λ*. The transformation matrices are calculated as below:

(13a) $$T_{r}=\left[\begin{array}{ccc} c\theta_{r} & s\theta_{r} & l_{r2}*s\theta_{r}+l_{r1}*s\theta_{r1}\\ -s\theta_{r} & c\theta_{r} & l_{r2}*c\theta_{r}+l_{r1}*c\theta_{r1}\\ 0 & 0 & 1 \end{array}\right]$$

(13b) $$T_{c}=\left[\begin{array}{ccc} c\theta_{c} & s\theta_{c} & r_{c}*\left(1-c\theta_{c}\right)\\ -s\theta_{c} & c\theta_{c} & r_{c}*s\theta_{c}\\ 0 & 0 & 1 \end{array}\right]$$

(13c) $$T_{e}=\left[\begin{array}{ccc} c\theta_{e} & s\theta_{e} & \int_{0}^{l_{e}}s(k(\lambda )*\lambda )d\lambda \\ -s\theta_{e} & c\theta_{e} & \int_{0}^{l_{e}}c(k(\lambda )*\lambda )d\lambda \\ 0 & 0 & 1 \end{array}\right]$$

#### 2) Experimental Set-up

**Fig. 7:**
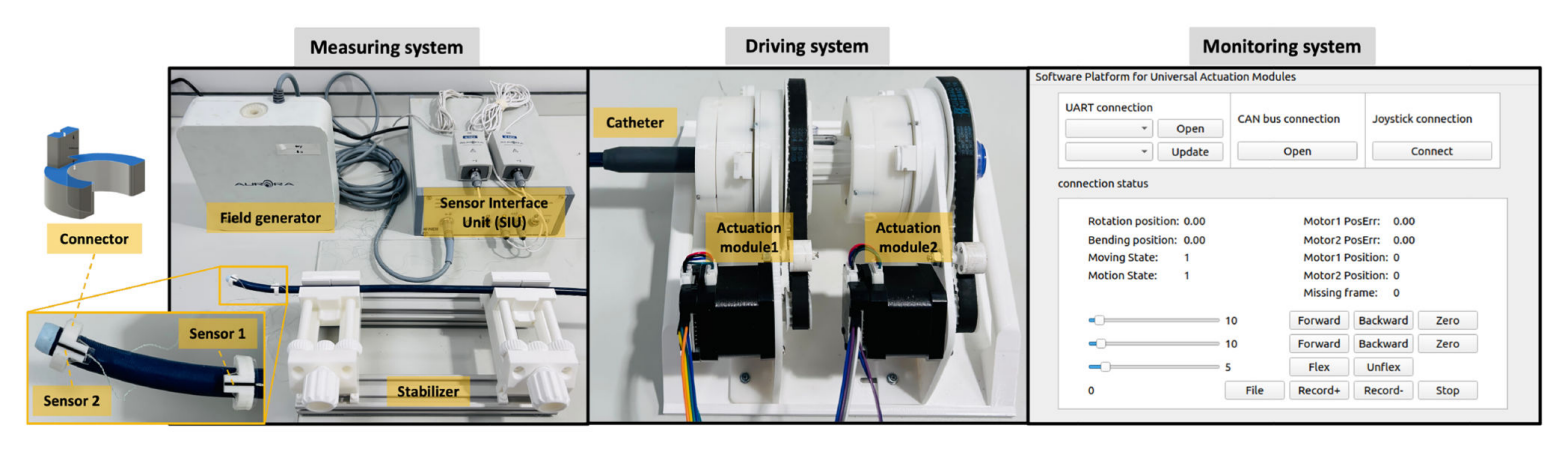
Components of the experimental set-up. The experimental arrangement comprises three distinct systems: software monitoring part for command transmission and feedback reception; driving part consisting of Module 1 designed for catheter handle gripping and Module 2 responsible for controlling the bending knob; An NDI electromagnetic tracking system serves as the measuring part. A connector is employed to establish a connection between the 6 DoF sensor and the catheter tip.

The experimental platform was established (Fig. 7) to measure two datasets: ${\bar{\theta }}_{k}$ (actuation angle) and ${\;}_{p}^{d}\bar{T}$ (the pose of the distal end relative to the proximal end of the BS) for the regression of model parameters in (11) and (12). Then the position (distance between model and BS tip) and direction (bending angle) errors were calculated by comparing ${\;}_{p}^{d}\bar{T}$ and *T*_*TC*−*PJ*_. The platform was composed of monitoring, driving, and measuring systems. ${\bar{\theta }}_{k}$ could be acquired directly from motor encoders in the driving system involving the use of two actuation modules to clamp the catheter in place and two vices for gripping the shaft of the catheter to simulate the working status of catheters in vessels. In the measuring system, 6 DoF electromagnetic (EM) sensors, a sensor interface unit and a field generator (NDI Aurora) were employed to track the BS poses. Customized connectors were designed to connect two EM sensors with the BS. ${\;}_{p}^{d}\bar{T}$ is the transformation matrix from sensor 2 to sensor 1. Furthermore, the user interface enabled the storage of datasets automatically.

To validate the universality of the kinematic model, we conducted experiments using five different tip sizes, each with distinct specifications as outlined in Table I. During these experiments, the knobs were driven to rotate 18 degrees (a sample size of around 55 values within the bending angle range which is enough for regression analysis) with a 5-second interval. The datasets were stored every second automatically for synchronization. Degrees of polynomials were chosen properly based on the patterns and trends observed within the datasets. The results are detailed in Section III.

## III Results

### A Random Planar Bending Simulation

**Fig. 8:**
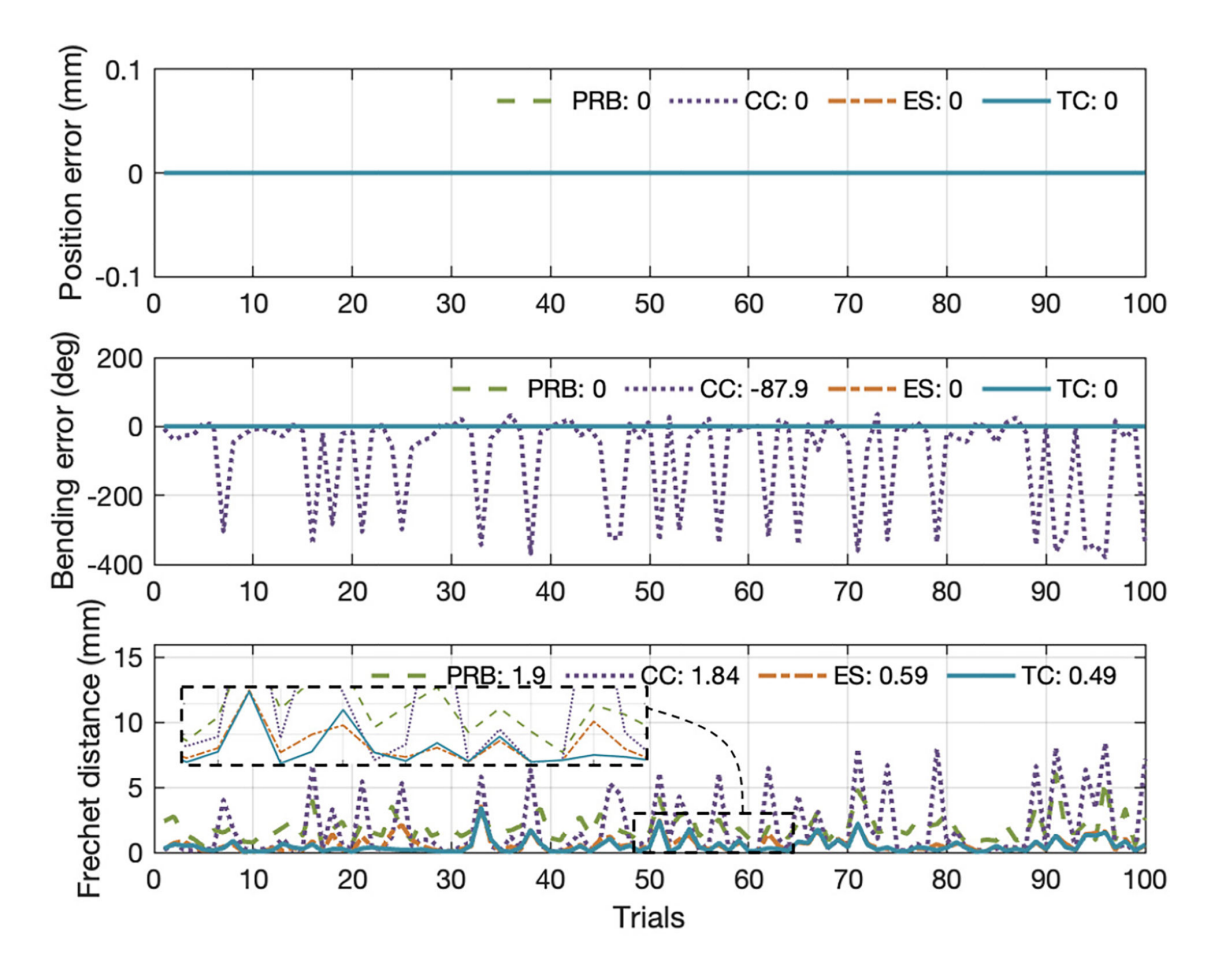
Modeling errors in one hundred trials. In these trials, all models could reach a randomly positioned target with varying bending angles, except for the CC model, which had an average bending error of -87.9 degrees. The average Fréchet distances for the PRB, CC, ES, and TC models were recorded as 1.90 mm, 1.84 mm, 0.59 mm, and 0.49 mm, respectively.

100 trials were conducted in the random planar bending simulation. The average tip position, bending angle errors, and Fr*é*chet distances of each model compared with the reference curve are summarized in Fig. 8. The position errors of the four models were zero, which means these catheter models can effectively reach any arbitrary tip positions. Moreover, they could match various bending angles exactly except for the CC model which showed a mean error of -87.9 degrees. It didn’t hold universally for BS with various curvatures. The PRB model was simplified and computationally tractable, but it lacked the requisite fidelity to capture the catheter shape with an average Fréchet distance of 1.90 mm. Additionally, the TC model demonstrated performance with an average Fréchet distance of only 0.49 mm, while the next best model (the ES model, characterized by its smoothly and continuously changing curvature) had a slightly higher distance of 0.59 mm. Hence, a TC model could be effectively employed to represent the characteristics of random planar bending and elongation of the BS compared with the other three models.

### B Bending Experiments

#### 1) Mapping from θ_k_ to$\theta_{p}^{\prime }$

**Fig. 9:**
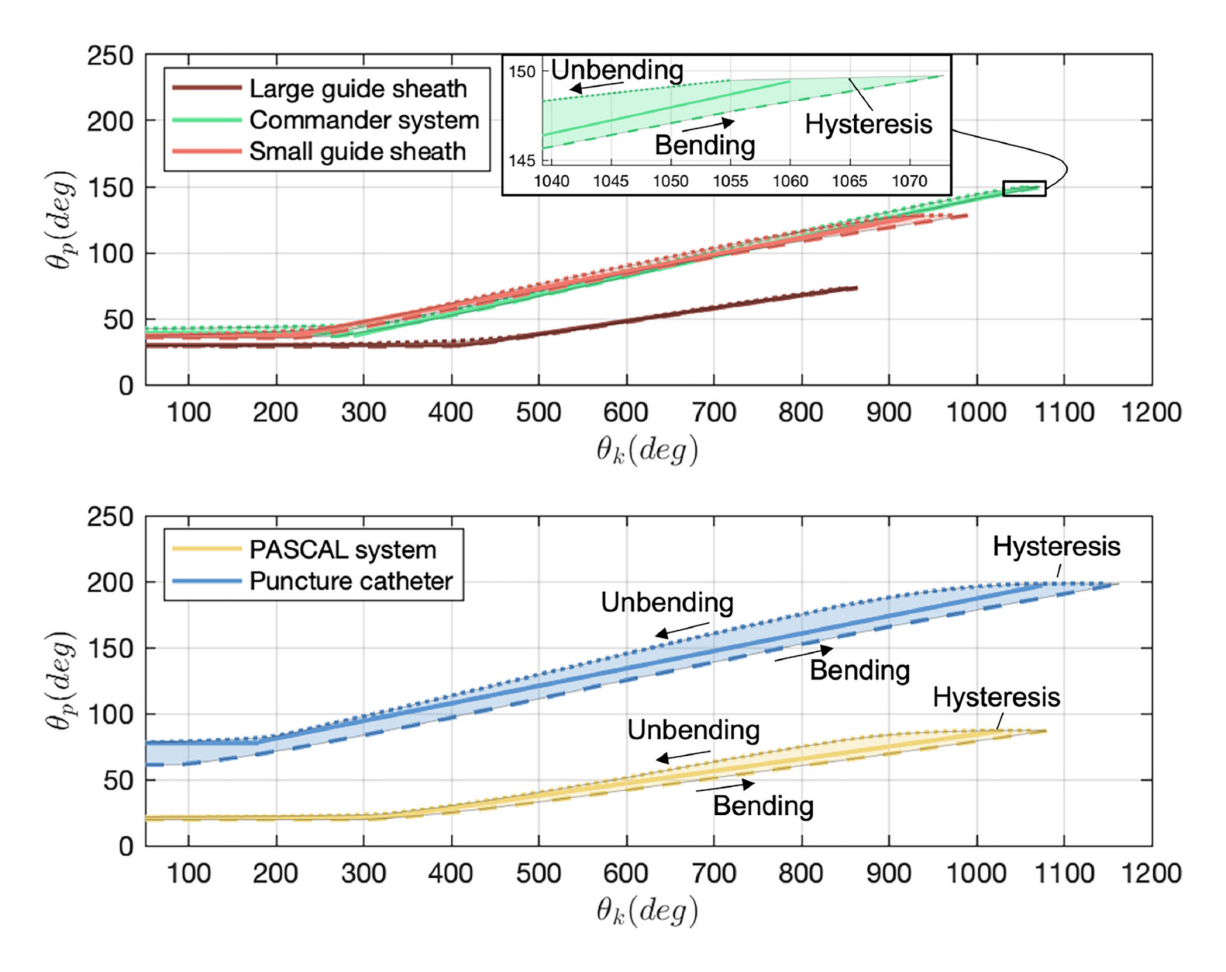
Regression analysis between the actuation module angle *θ*_*k*_ and the bending angle $\theta_{p}^{\prime }$.

Before calculating the tip poses, we analysed the regression from *θ*_*k*_ to $\theta_{p}^{\prime }$. The experimental data of the five catheters, illustrated in Fig. 9 as dashed lines for bending and dotted lines for unbending, exhibited a common trend called a dead zone ([0, Θ_*k*_)) at the initial phase. In addition, there existed a difference (hysteresis) between the bending and unbending patterns mainly due to friction and transmission looseness, especially in the puncture catheter and PACSAL system. Based on the trends of the data, we decided to divide the regression analysis into two distinct sections to deal with the dead zone. These sections were characterized by fittings involving both constant values *A* and first-order polynomials *B*, as demonstrated by the solid line in the figure. The hysteresis could be compensated by imposing additional input compensator *δB* to the *B* during the manipulation, as shown in (14).

(14) $$\theta_{p}^{\prime }=\{\begin{array}{ll} A, & \text{if}\;0\leq \theta_{k}<{\text{Θ}}_{k}\\ (B\pm \delta B)*\left(\theta_{k}-{\text{Θ}}_{k}\right)+A, & \text{if}\;{\text{Θ}}_{k}\leq \theta_{k} \end{array}$$

#### 2) Mapping from $\theta_{p}^{\prime }$ to T_TC−PJ_

**Fig. 10:**
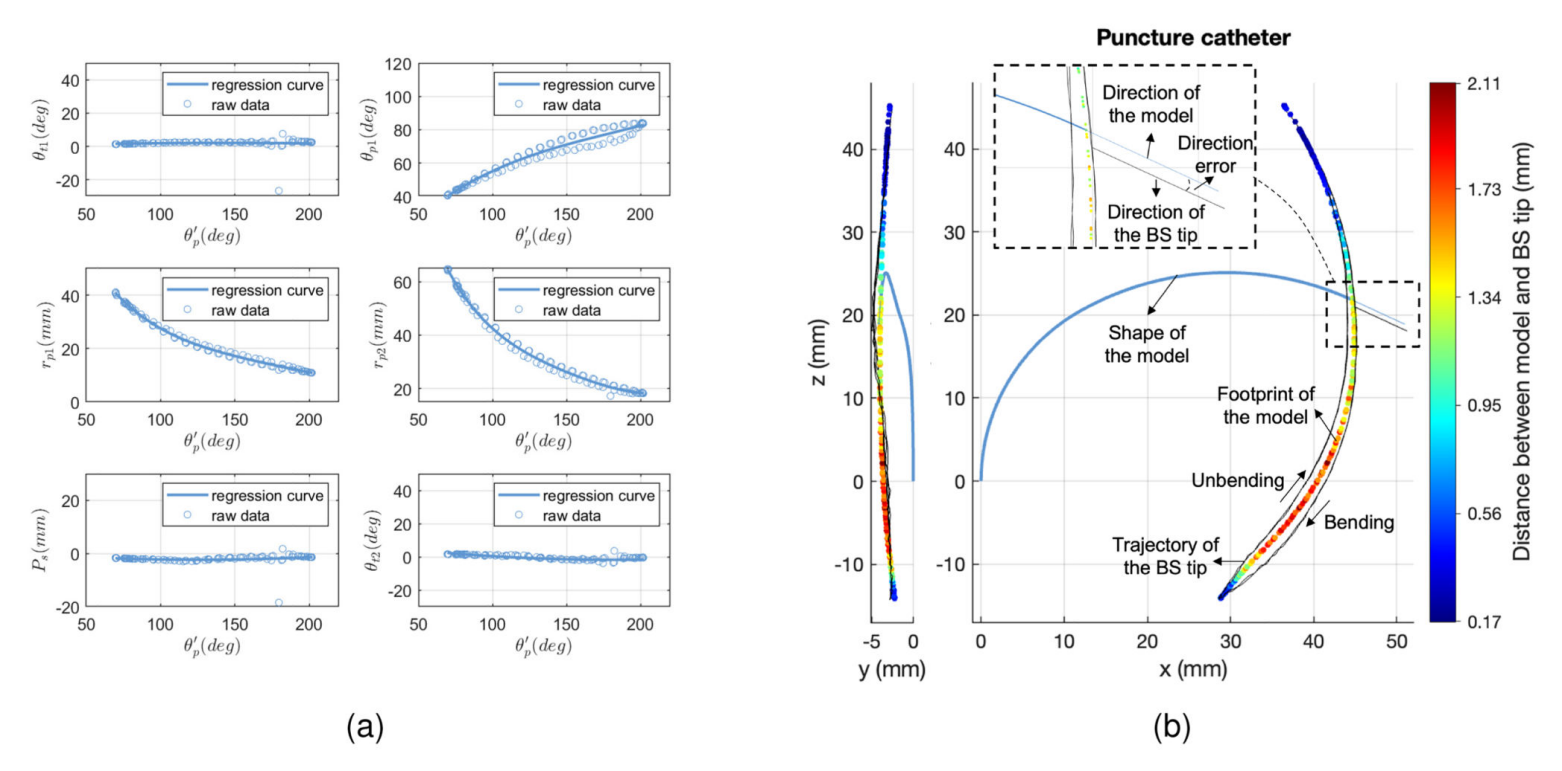
Regression and positioning analysis of the puncture catheter. (a) Regression analysis from the bending angle $\theta_{p}^{\prime }$ to the model variables. The results are obtained through the puncture catheter, with a regression methodology applicable across various catheters. (b) Positioning analysis including position and direction errors.

For the mapping from $\theta_{p}^{\prime }$ to the TC-PJ model variables, we identified that the same polynomial methodology could be applied consistently across all catheters, so, we took the puncture catheter as an example. The regression results of it are visually represented in Fig. 10a. The ranges of pseudo joints *θ*_*t*1_, *P*_*s*_, and *θ*_*t*2_ exhibited relatively stable behaviour around zero, indicating that the catheter BS bent nearly in a plane experiencing very small torsion and shear strain deformation patterns. Conversely, the regression error was notably influenced by TC model parameters *θ*_*p*1_, *r*_*p*1_, and *r*_*p*2_. Consequently, we have determined that polynomial degrees of 3, 4, and 4 offer the best performance for modeling these variables. While increasing the polynomial degrees could reduce the error, it came at an increased computational cost, necessitating a balance between accuracy and efficiency. Based on the regression outcome and TC-PJ model, the mapping from $\theta_{p}^{\prime }$ to *T*_*TC*−*PJ*_ was calculated, and the corresponding outcomes are presented in Fig. 10b. The catheter BS was bending in the x-z plane and also moved in the y direction because of torsion and shear strain. The hysteresis was getting larger when reaching the bending angle range because of the tension along the catheter, leading to larger position errors. Using the same methods, we extended our analysis to the remaining four catheters.

We have done the bending and unbending experiments twice for each catheter. The BS tip position and direction root-mean-square error (RMSE) and standard deviation (SD) are summarized in Table II. Regardless of some random errors, there is a small difference between bending and unbending, the first trial and the second trial. The puncture catheter exhibited the highest errors, with position and direction RMSE (SD) extending to around 1.16 (0.53) mm and 0.70 (31.33) degrees, and the large error particularly happens when the bending angle exceeded 90 degrees. These errors were primarily attributed to the mechanism coupling of two sequential bendable components. In contrast, the large guide sheath (with the highest stiffness) and the PASCAL^*TM*^ system (with the shortest bendable length) demonstrated a consistently stable performance, characterized by low RMSE (SD) of around 0.10 (0.051) mm and 0.049 (2.15) degrees, and 0.31 (0.15) mm and 0.14 (6.14) degrees, respectively. The small guide sheath and the Commander^*TM*^ system exhibited intermediate performance, with position RMSE (SD) of around 0.85 (0.40) mm and direction RMSE (SD) of 0.25 (9.80) degrees. It is important to note that the catheters were collected after the procedures and had undergone permanent deformation, contributing to the observed differences in performance significantly.

## IV Discussions and Conclusions

Our study introduces a universal actuation module designed for the manipulation of commercial heart valve interventional catheters, along with a universal kinematic model that combines a two-curvature model with pseudo joints for precise BS positioning. The TC model was first studied in a planar bending simulation compared with three commonly used models (PRB, CC, and ES), and it showed a more accurate shape representation. Then the universality was evaluated by our established experimental setup. The actuation module showed a universal clamping performance for five catheters with a diameter lower than 45 mm produced by Edwards Lifesciences company. While adaptability to larger catheters and lateral devices remains a challenge due to spatial constraints, this limitation can be addressed through adjustments in size. Furthermore, the TC-PJ model exhibited promising adaptability and free-space positioning precision for commercial catheters with an average tip position and direction RMSE of 0.65 mm and 0.23 degrees in free-space. Notably, the error is mainly influenced by the regression process. We advocate choosing specific degrees of the polynomial regression based on the application, which is a balance between control accuracy and response. Future work will integrate actuation modules with rotational and translational mechanisms to enable comprehensive control of catheters.

## Figures and Tables

**Table I:** Five Experimental Catheters from Edwards Lifesciences

Bendable tips			
Catheters	L (mm)	D (Fr)	Setup
Sheath (large)	50	30	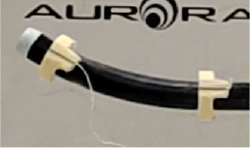
PASCAL*™*	28	17	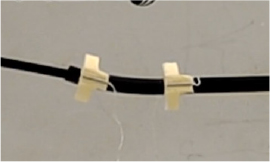
Commander*™*	100	16	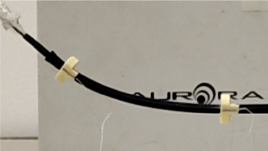
Puncture	65+23	18	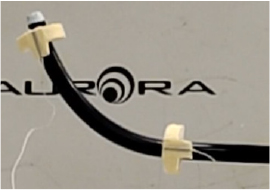
Sheath (small)	42	22	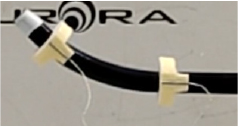

**Table II:** Bs Tip Position and Direction RMSE (SD) of Five Catheters

Trials	Position RMSE (SD) (mm)				Direction RMSE (SD) (deg)			
	Bending 1	Unbending 1	Bending 2	Unbending 2	Bending 1	Unbending 1	Bending 2	Unbending 2
Sheath (large)	0.093 (0.050)	0.091 (0.049)	0.10 (0.051)	0.098 (0.057)	0.044 (2.07)	0.045 (1.88)	0.049 (2.15)	0.039 (1.73)
PASCAL*™*	0.33 (0.13)	0.30 (0.14)	0.28 (0.17)	0.31 (0.15)	0.21 (9.37)	0.14 (6.14)	0.13 (6.15)	0.13 (6.02)
Commander*™*	1.22 (0.38)	0.84 (0.35)	0.81 (0.35)	0.89 (0.32)	0.31 (14.33)	0.19 (9.16)	0.25 (9.78)	0.29 (13.66)
Puncture	1.16 (0.51)	1.22 (0.60)	1.15 (0.52)	1.16 (0.53)	0.70 (31.33)	0.49 (24.40)	0.50 (24.98)	0.47 (23.52)
Sheath (small)	0.73 (0.27)	0.82 (0.44)	0.74 (0.35)	0.81 (0.41)	0.14 (6.28)	0.15 (6.28)	0.17 (7.71)	0.15 (7.49)
